# Contraction stimulates muscle glucose uptake independent of atypical PKC

**DOI:** 10.14814/phy2.12565

**Published:** 2015-11-12

**Authors:** Haiyan Yu, Nobuharu L. Fujii, Taro Toyoda, Ding An, Robert V. Farese, Michael Leitges, Michael F. Hirshman, Joram D. Mul, Laurie J. Goodyear

**Affiliations:** ^1^ Harvard Medical SchoolJoslin Diabetes CenterBostonMassachusetts; ^2^ James A. Haley Veterans Medical CenterTampaFlorida; ^3^ The Biotechnology Center of OsloUniversity of Oslo, BlindernOsloNorway

**Keywords:** Glucose uptake, atypical PKC, Muscle contraction, Contraction‐stimulated glucose uptake

## Abstract

Exercise increases skeletal muscle glucose uptake, but the underlying mechanisms are only partially understood. The atypical protein kinase C (PKC) isoforms *λ* and *ζ* (PKC‐*λ*/*ζ*) have been shown to be necessary for insulin‐, AICAR‐, and metformin‐stimulated glucose uptake in skeletal muscle, but not for treadmill exercise‐stimulated muscle glucose uptake. To investigate if PKC‐*λ*/*ζ* activity is required for contraction‐stimulated muscle glucose uptake, we used mice with tibialis anterior muscle‐specific overexpression of an empty vector (WT), wild‐type PKC‐*ζ* (PKC‐*ζ* ^WT^), or an enzymatically inactive T410A‐PKC‐*ζ* mutant (PKC‐*ζ* ^T410A^). We also studied skeletal muscle‐specific PKC‐*λ* knockout (M*λ* KO) mice. Basal glucose uptake was similar between WT, PKC‐*ζ* ^WT^, and PKC‐*ζ* ^T410A^ tibialis anterior muscles. In contrast, in situ contraction‐stimulated glucose uptake was increased in PKC‐*ζ* ^T410A^ tibialis anterior muscles compared to WT or PKC‐*ζ* ^WT^ tibialis anterior muscles. Furthermore, in vitro contraction‐stimulated glucose uptake was greater in soleus muscles of M*λ* KO mice than WT controls. Thus, loss of PKC‐*λ*/*ζ* activity increases contraction‐stimulated muscle glucose uptake. These data clearly demonstrate that PKC‐*λ*/*ζ* activity is not necessary for contraction‐stimulated glucose uptake.

## Introduction

Normal regulation of glucose transport into skeletal muscle in response to physical exercise or postprandial insulin stimulation is critical for the maintenance of whole‐body glucose homeostasis (Richter and Hargreaves 2013; Schwartz et al. 2013). The underlying mechanisms mediating insulin‐stimulated glucose transport have been largely elucidated and are thought to involve a complex signaling cascade that includes the insulin receptor tyrosine kinase, phosphatidylinositol‐3‐kinase, Akt, and the RabGAP proteins TBC1D4 (also known as AS160) and TBC1D1, that eventually leads to the movement of glucose transporter 4 (GLUT4) to the cell surface (Biddinger and Kahn 2006; Taylor et al. 2008). While exercise and muscle contractile activity also cause GLUT4 translocation, it is well established that the initiating signaling molecules that mediate this process are distinct from that of insulin (Ploug et al. 1987; Constable et al. 1988; Douen et al. 1990; Hayashi et al. 1998; Lemieux et al. 2000). The signaling proteins that regulate exercise‐ and contraction‐stimulated glucose uptake are still not clearly understood, and there is considerable evidence that redundant signaling mechanisms may control this important physiological process (Rockl et al. 2008).

AMP‐activated protein kinase (AMPK), a master regulator of cellular energy homeostasis, has been proposed to be the central node regulating glucose transport in response to insulin‐independent stimuli such as exercise, muscle contraction, hypoxia, metformin, and the AMPK activator AICAR (Merrill et al. 1997; Mu et al. 2001; Sajan et al. 2010; Hardie 2011; Richter and Hargreaves 2013). A number of studies using different animal models have convincingly demonstrated that AMPK is necessary for AICAR‐ and metformin‐stimulated glucose transport (Zhou et al. 2001; Fryer et al. 2002; Sajan et al. 2010). In contrast, while some studies have shown that AMPK is important for contraction‐stimulated glucose transport (Mu et al. 2001; Jensen et al. 2008), many other studies have revealed minimal or no reduction in contraction‐induced muscle glucose uptake in AMPK loss‐of‐function mouse models (Jorgensen et al. 2004; Fujii et al. 2005; Witczak et al. 2007; Merry et al. 2010). Similarly, human studies have also suggested that AMPK does not play a key role in muscle substrate combustion during exercise training (Wojtaszewski et al. 2002; McConell et al. 2005; Mortensen et al. 2013). Furthermore, muscle‐specific knockout of LKB1 (liver kinase B1), the kinase upstream of AMPK in skeletal muscle, only partially decreases contraction‐stimulated muscle glucose uptake (Sakamoto et al. 2005; Koh et al. 2006). These data suggest that additional signaling mechanisms must contribute to contraction‐stimulated glucose uptake in skeletal muscle.

Atypical protein kinase C (aPKC) isoforms are members of the PKC family of serine/threonine kinases and include the *λ*/*ι* (aPKC‐*λ* is the mouse homolog of aPKC‐*ι*) and *ζ* isoforms (Newton 2001; Farese et al. 2014). PKC‐*λ* is the major aPKC in murine muscle (Akimoto et al. 1994; Sajan et al. 2006; Farese et al. 2007), and has approximately 70% similarity with PKC‐*ζ* (Akimoto et al. 1994; Standaert et al. 1999). In rodents, PKC‐*ζ* and PKC‐*λ* show a high degree of redundancy in the regulation of glucose transport (Bandyopadhyay et al. 1999a; Sajan et al. 2006; Farese et al. 2007; Habets et al. 2012) and are often referred to as PKC‐*λ*/*ζ*. Muscle‐specific PKC‐*λ* knockout (M*λ*KO) mice have impaired insulin‐stimulated glucose uptake in the vastus lateralis muscle and heart in vivo (Farese et al. 2007). Contrary to these findings, stable depletion of PKC‐*λ* in L6 myotubes using a lentiviral shRNA approach resulted in enhanced insulin sensitivity and glucose uptake (Stretton et al. 2010). In humans, PKC‐*λ*/*ζ* activity is impaired in obese, prediabetic, and diabetic subjects (Vollenweider et al. 2002; Beeson et al. 2003, 2004; Kim et al. 2003; Bandyopadhyay et al. 2005). Thus, understanding the role of PKC‐*λ*/*ζ* activity during muscle glucose uptake has important physiological and clinical relevance.

Exercise causes a modest increase in aPKC activity and/or phosphorylation in both rodents (Chen et al. 2002; Aschenbach et al. 2006) and humans (Perrini et al. 2004; Richter et al. 2004), and PKCs have long been proposed to play a role in the regulation of exercise‐stimulated glucose transport (Cleland et al. 1990; Ihlemann et al. 1999a). Using M*λ*KO mice, PKC‐*λ* activity was shown to be required for stimulation of glucose uptake with the AMPK activators AICAR and metformin (Sajan et al. 2010). In contrast, treadmill running exercise, which also causes AMPK activation, resulted in normal increases in glucose transport in this genetic mouse model (Sajan et al. 2010). It is not known if more intense forms of exercise, such as tetanic muscle contractions, require PKC‐*λ*. In addition, the putative role of PKC‐*ζ* in contraction‐stimulated glucose transport has not been investigated. In the current study, we investigated the roles of both PKC‐*λ* and PKC‐*ζ* in contraction‐stimulated glucose uptake in skeletal muscle. For this purpose, we studied mice with expression of an enzymatically inactive T410A‐PKC‐*ζ* mutant selectively in the tibialis anterior (TA) muscle, as well as M*λ*KO mice.

## Experimental Methods

### Protocols

The Institutional Animal Care and Use Committee at the Joslin Diabetes Center reviewed and approved all animal protocols.

### Mice

Eight‐week‐old female ICR mice (Taconic, Hudson, NY) were used for in vivo gene transfer and expression. PKC‐*λ* ^flox/flox^; MCK‐*Cre* ^−/−^ and PKC‐*λ* ^flox/+^; MCK‐*Cre* ^+/−^ mice on a C57Bl/6 background (Farese et al. 2007) were bred to generate littermate wild‐type (WT) control and homozygous M*λ*KO mice (9–11 weeks old). Genotyping was done as previously described (Farese et al. 2007). Mice were maintained in a light‐ and temperature‐controlled environment (12 h light/dark, 20–24°C) and had ad libitum access to water and standard mouse diet (21% kcal from fat; 9F 5020 Lab Diet, PharmaServ Inc.).

### Materials

[^32^P]‐ATP (PerkinElmer, Boston, MA) was used for PKC‐*λ*/*ζ* activity assays. WT‐PKC‐*ζ* and T410A‐PKC‐*ζ* (this mutation prevents phosphorylation at Thr^410^) constructs have been described previously (Standaert et al. 1999).

### In situ contraction and glucose uptake

Plasmid DNA (Standaert et al. 1999) was extracted and purified using a QIA filter Plasmid Kit from Qiagen (Valencia, CA). Enriched yields of plasmid DNA were directly injected into mouse TA muscles followed by electroporation as described previously (Fujii et al. 2004; Ho et al. 2004; Yu et al. 2004). Seven days after gene delivery, mice were subjected to in situ muscle contraction and glucose uptake was measured (Ho et al. 2004; Kramer et al. 2006). Briefly, mice were anesthetized and peroneal nerves of both legs were surgically exposed, and electrodes were attached and subjected to electrical stimulation for 15 min (train rate: 1/sec; train duration: 500 msec; pulse rate: 100 Hz; duration: 0.1 msec at 2–5 V). Legs not stimulated to contract (basal) were used as sham‐operated controls. Immediately after nerve stimulation, TA muscles were rapidly removed, frozen in liquid nitrogen, and used for western blot analyses or activity assays (see below). Other mice were used to measure in situ glucose uptake, as described previously (Ho et al. 2004; Kramer et al. 2006). Briefly, basal tail vein blood samples were collected prior to intravenous bolus delivery of [^3^H]‐2‐deoxyglucose in saline (PerkinElmer Life Sciences) through the retro‐orbital sinus (Yardeni et al. 2011). During contraction studies, the tracer bolus was injected simultaneous to the onset of in situ peroneal nerve stimulation. Tail vein blood samples were taken 5, 10, 15, 25, 35, and 45 min postinjection to determine blood glucose‐ and [^3^H]‐2‐deoxyglucose‐specific activity. TA muscles were removed after mice were euthanized and quickly frozen in liquid nitrogen. Subsequently, the muscle tissue was processed to determine the accumulation rate of [^3^H]‐2‐deoxyglucose, as previously described (Ho et al. 2004).

### Contraction and glucose uptake of isolated muscles

Soleus muscles from WT and M*λ*KO mice were isolated, adjusted to maintain resting length and tension, as previously described (Cerletti et al. 2008; Rowe et al. 2013; Sinha et al. 2014), and were incubated for 30 min at 37°C in Krebs–Ringer bicarbonate (KRB) buffer containing 2 mmol/L pyruvate. Muscles were contracted by electrical stimulation (train rate: 2/min, train duration: 10 sec, pulse rate: 100 Hz, duration: 0.1 msec at 100 V) for 10 min, and force production was monitored using an isometric force transducer (Kent Scientific, Litchfield, CT) with the converted digital signal captured by a data acquisition system (iWorx114; CB Sciences, Dover, NH) and analyzed with software (Labscribe; CB Sciences). Glucose uptake in isolated muscles was measured as described previously (Wojtaszewski et al. 1999; Yu et al. 2006).

### Muscle processing

TA muscle samples (PKC‐*ζ* experiments) were isolated, and gene transfer and protein expression were confirmed in muscle lysates by western blot analyses. Muscle lysates were prepared as described previously (Aschenbach et al. 2006). Soleus muscle samples (M*λ*KO mice) were used for western blot analyses, electrophoresis separation of myosin heavy chain isoforms (Talmadge and Roy 1993), citrate synthase activity (Srere 1969), and aPKC activity (see below).

### Atypical PKC activity

Atypical PKC activity was measured as described previously (Farese et al. 2007). Briefly, aPKCs were immunoprecipitated with polyclonal anti‐PKC‐*λ*/*ζ* antibody, collected on Sepharose‐Protein A/G beads, and incubated for 8 min at 30°C with [*γ*‐^32^P]‐ATP and the serine analog of the PKC‐*ε* pseudosubstrate (BioSource). After incubation, ^32^P‐labeled substrate was trapped on P‐81 filter paper, washed, dried, and counted in a liquid scintillation counter.

### Western blot analyses

Western blot analyses were done as described previously (Aschenbach et al. 2006; Farese et al. 2007). In short, muscle lysates were immunoblotted for PKC‐*λ*/*ζ* (rabbit polyclonal antiserum; Santa Cruz Biotechnology Inc.), which recognizes C‐termini of both aPKCs and pPKC‐*λ*/*ζ* (Thr^403/410^). Rabbit polyclonal antiserum for phospho‐AMPK‐Thr^172^, AMPK*α*2, and AS160/TBC1D1 PAS site phosphorylation were from Cell Signaling Technology (Danvers, MA).

### Statistical analysis

Data are presented as mean ± SEM. Statistical significance was determined by one‐way, two‐way, or repeated measures ANOVA with Tukey's HSD post hoc analysis, or by unpaired Student's *t*‐test. Differences were considered significant when *P *<* *0.05.

## Results

### Overexpression of PKC‐ζ^WT^ and PKC‐ζ^T410A^ in mouse TA muscles

**Figure 1:**
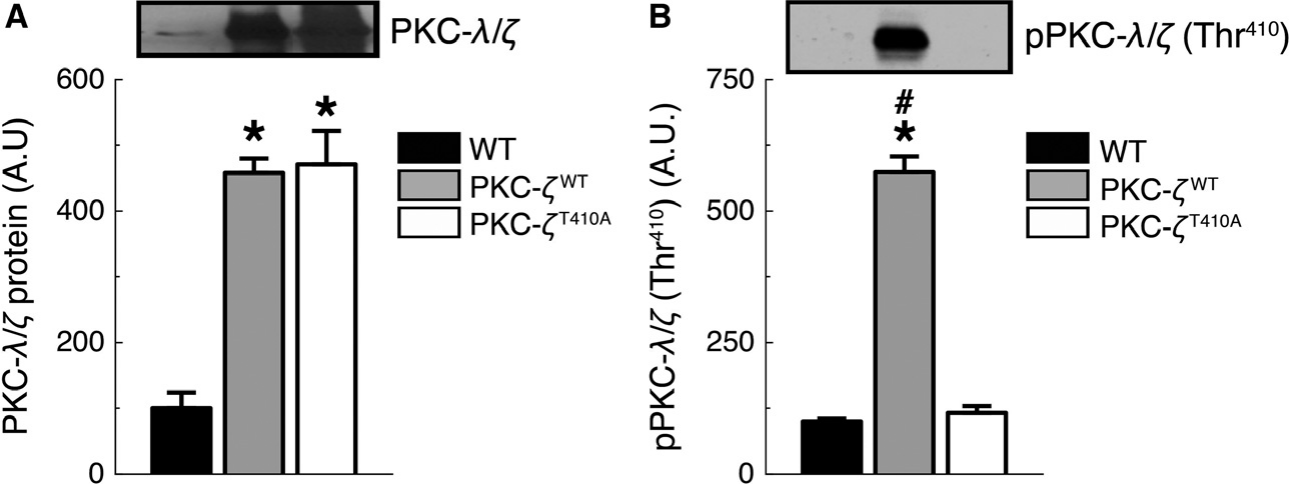
Overexpression of PKC‐*ζ* ^WT^ and PKC‐*ζ* ^T410A^ in mouse TA muscles. Representative images (top) and immunoblot analyses of (A) PKC‐*λ*/*ζ* protein levels (*n *=* *6–9/group) and (B) pPKC‐*λ*/*ζ* (Thr^410^) levels (*n *=* *4–5/group) in WT, PKC‐*ζ* ^WT^, and PKC‐*ζ* ^T410A^ TA muscles. *P <* *0.001, versus WT; #*P *<* *0.001, versus PKC‐*ζ* ^T410A^.

**Figure 2:**
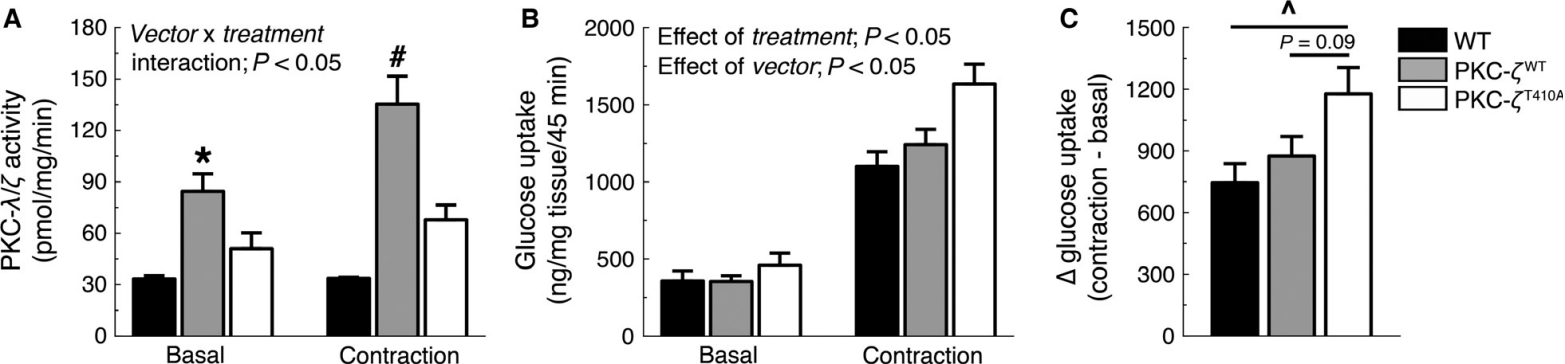
PKC‐*λ*/*ζ* activity and glucose uptake in PKC‐*ζ* ^WT^ and PKC‐*ζ* ^T410A^ TA muscles. (A) Basal and contraction‐stimulated PKC‐*λ*/*ζ* activity (*n *=* *3–5/group) (B) in situ basal and contraction‐stimulated glucose uptake (*n *=* *5–9/group), and (C) ∆ glucose uptake (contraction minus basal) in WT, PKC‐*ζ* ^WT^, and PKC‐*ζ* ^T410A^ TA muscles. *P <* *0.05, versus WT‐Basal, WT‐Contraction, PKC‐*ζ* ^WT^‐Contraction; ^#^ *P *<* *0.05, versus all other groups; ^^^ *P *<* *0.05, versus WT.

To investigate PKC‐*ζ* function during contraction‐stimulated muscle glucose uptake, we overexpressed an empty vector (wild type; WT), a vector containing WT PKC‐*ζ* (PKC‐*ζ* ^WT^), or a vector containing the dominant‐negative T410A‐PKC‐*ζ* mutant (PKC‐*ζ* ^T410A^) in mouse TA muscles. The T410A mutation prevents phosphorylation at Thr^410^ and renders the kinase inactive (Bandyopadhyay et al. 1999b). Seven days following gene transfer and electroporation, protein levels of immunoreactive aPKC (PKC‐*λ* plus PKC‐*ζ*) were fivefold higher in TA muscles expressing PKC‐*ζ* ^WT^ and PKC‐*ζ* ^T410A^ compared to WT muscles (Fig. 1A). We used an antibody that detects general aPKC levels (i.e. PKC‐*λ* plus PKC‐*ζ*) as these proteins show a high degree of redundancy in the regulation of glucose transport (Bandyopadhyay et al. 1999a; Sajan et al. 2006; Farese et al. 2007; Habets et al. 2012) and are often referred to as PKC‐*λ*/*ζ*. PKC‐*ζ* ^WT^ overexpressing muscles had greater pPKC‐*ζ* (Thr^410^) levels compared to WT and PKC‐*ζ* ^T410A^ muscles (Fig. 1B). The fivefold increase in aPKC levels in PKC‐*ζ* ^T410A^ muscles, with no increase in phosphorylation, demonstrates the effectiveness of the mutation. Under basal conditions, aPKC activity was significantly higher in muscles expressing PKC‐*ζ* ^WT^ compared to WT muscles (Fig. 2A). Atypical PKC activity in muscles expressing PKC‐*ζ* ^T410A^ was not significantly different from PKC‐*ζ* ^WT^ or WT muscles (Fig. 2A). In situ muscle contraction increased aPKC activity in PKC‐*ζ* ^WT^ muscles but did not significantly increase aPKC activity in PKC‐*ζ* ^T410A^ or WT muscles (Fig. 2A).

### Contraction‐stimulated glucose uptake in PKC‐ζ^WT^ and PKC‐ζ^T410A^ TA muscles

We next determined the effects of altered PKC‐*ζ* activity on basal and contraction‐stimulated glucose uptake in the TA muscles. Under basal conditions, there was no difference in rates of glucose uptake among the different treatments. Contraction increased glucose uptake in all groups (Fig. 2B). Interestingly, muscles overexpressing the mutant PKC‐*ζ* ^T410A^ had a significantly greater increase in contraction‐stimulated glucose uptake (Fig. 2B and C). These data demonstrate that decreased PKC‐*ζ* activity increases contraction‐stimulated glucose uptake in skeletal muscle.

### Contraction‐stimulated glucose uptake in MλKO mice

**Figure 3:**
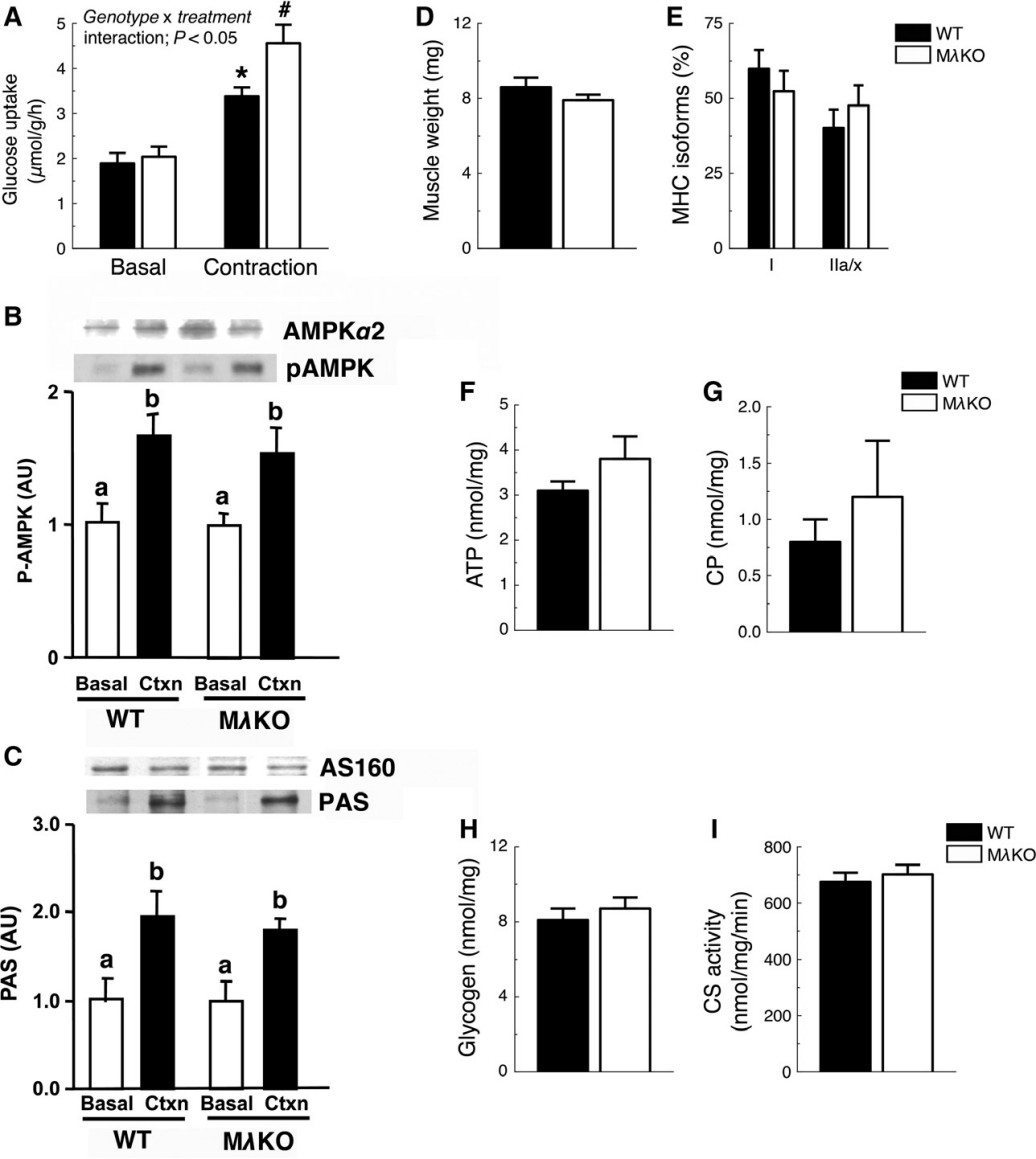
Contraction‐mediated glucose uptake and function in soleus muscles of M*λ* KO mice. (A) Basal and contraction‐induced in vitro glucose uptake (*n *=* *7–12/group) (B) muscle weight (C) relative proportion of myosin heavy chain (MHC) isoforms I and IIa/x (D) adenosine triphosphate (ATP), (E) creatine phosphate (CP), (F) glycogen levels, and (G) citrate synthase (CS) activity in soleus muscles of WT and M*λ* KO mice (*n *=* *3–6/group). (H) Phospho‐AMPK (pAMPK) and (I) PAS phosphorylation levels in soleus muscles during basal and contraction (*n *=* *5/group). *P <* *0.05, versus WT‐Basal, M*λ* KO‐Basal; ^#^ *P *<* *0.05, versus all other groups. Different letters indicate significant difference as following: ^a,b^ *P *<* *0.05, effect of *treatment*.

We next focused on PKC‐*λ*, studying knockout mice with muscle‐specific deletion of PKC‐*λ* (M*λ*KO). These mice have an approximately 80% reduction in total aPKC expression and activity in skeletal muscle (Farese et al. 2007). Basal rates of muscle glucose uptake in soleus muscles were similar between WT littermate control mice and M*λ*KO mice (Fig. 3A). Muscle contraction in isolated soleus muscles increased in vitro muscle glucose uptake in both genotypes (Fig. 3A). However, contraction‐stimulated glucose uptake in soleus muscles was significantly higher in M*λ*KO mice compared to WT controls (Fig. 3A). Thus, using two different models we demonstrate that loss of aPKC activity increases contraction‐stimulated muscle glucose uptake.

### Muscle signaling and physiology in MλKO mice

To assess whether the increased contraction‐mediated muscle glucose uptake in M*λ*KO mice is associated with changes in contraction‐stimulated signaling proteins that mediate glucose uptake, we performed immunoblots for phospho‐AMPK and phospho‐AKT substrate (PAS) site phosphorylation of AS160/TBC1D1 (Taylor et al. 2008). Contraction similarly increased phospho‐AMPK and AS160/TBC1D1 PAS site phosphorylation in soleus muscles of WT and M*λ*KO mice (Fig. 3B and C), indicating that these signaling proteins likely are not responsible for the increased contraction‐mediated muscle glucose uptake phenotype in M*λ*KO mice. To determine if muscle‐specific knockdown of PKC‐*λ* alters other factors that influence muscle function, we measured muscle weights, myosin heavy chain isoforms I and IIa/x, adenosine triphosphate (ATP), creatine phosphate (CP), glycogen content, and citrate synthase activity in soleus muscles. There was no significant effect of muscle‐specific PKC‐*λ* knockdown on these measurements (Fig. 3D–I).

### Contraction force in soleus muscles of MλKO mice

**Figure 4:**
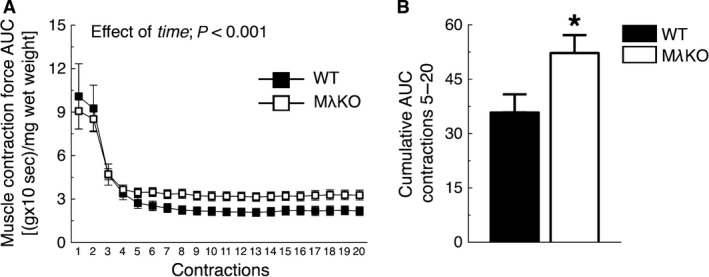
Contraction force in soleus muscles of M*λ* KO mice. (A) Contraction force area under the curve (AUC) during 20 subsequent contractions and (B) cumulative AUC muscle force production during contractions 5 till 20 in soleus muscles of WT and M*λ* KO mice (*n *=* *6–10/group). *P <* *0.05, versus WT.

We next tested the hypothesis that decreased aPKC activity would increase muscle contraction force, since force production is positively correlated with glucose uptake in skeletal muscle (Ihlemann et al. 1999b, 2001). In vitro force production was measured in soleus muscle from WT and M*λ*KO mice. Although force production was similar between groups during the initial five contractions, force production in the later contractions was higher in M*λ*KO mice compared to WT mice (Fig. 4A). Indeed, cumulative muscle force production during contractions 5 through 20 was significantly higher in M*λ*KO mice compared to WT mice (Fig. 4B). Thus, loss of aPKC activity appears to increase the muscle's resistance to fatigue. Considering our previous observations, it seems unlikely that this is due to changes in muscle fiber type, preserved energy, or mitochondrial mass.

## Discussion

The goal of this study was to determine if aPKC activity is necessary for contraction‐stimulated glucose uptake in skeletal muscle. We found that aPKC activity is not required for contraction‐induced glucose uptake, and in fact, impairment of aPKC activity resulted in significant improvements in contraction‐induced muscle glucose uptake. The increase in contraction‐stimulated glucose uptake was accompanied by an increased resistance to muscle fatigue. Because contraction‐induced muscle glucose uptake is associated with muscle force production and tension (Ihlemann et al. 1999b, 2001), our data indicate that the increased contraction‐induced muscle glucose uptake in M*λ*KO mice may be due, at least in part, to improved muscle contraction force. To date, aPKC activity has not been linked to muscle contraction force. The mechanism for the increase in contraction force is still not known, but we can rule out a role for muscle fiber composition, energy storage, or mitochondrial mass in soleus muscles, since none of these factors were changed in the M*λ*KO mice.

Exercise increases aPKC activity in mice and humans (Chen et al. 2002; Beeson et al. 2003; Richter et al. 2004; Aschenbach et al. 2006; Sajan et al. 2010), and PKCs have also been implicated in the regulation of glucose uptake in skeletal muscle (Cleland et al. 1990; Ihlemann et al. 1999a; Farese and Sajan 2010). Taken together, these findings suggest that aPKCs could function in the regulation of contraction‐ and exercise‐stimulated glucose uptake. However, previous work has shown that aPKCs are not required for treadmill exercise‐induced muscle glucose uptake (Sajan et al. 2010), which is consistent with our current study where functional disruption of both aPKC isoforms did not decrease contraction‐stimulated muscle glucose uptake. Thus, aPKC activity is not necessary for treadmill‐ (Sajan et al. 2010) or contraction‐induced (current study) glucose uptake in skeletal muscle. It is interesting that in contrast to exercise and contraction, stimulation of glucose uptake by AICAR and metformin, two activators of AMPK in skeletal muscle, do require aPKC for normal glucose uptake (Sajan et al. 2010). Exercise and muscle contraction are also well established and potent stimulators of AMPK (Witczak et al. 2008; Richter and Hargreaves 2013), but clearly we find that the AMPK‐aPKC signaling axis is not necessary for glucose uptake with these stimuli. The lack of effect of disrupting the aPKCs is consistent with the concept that the intracellular signaling mechanism that regulates contraction‐stimulated glucose uptake has a high degree of redundancy and is multifactorial, with one signaling pathway compensating when there is the loss of another signaling protein (Witczak et al. 2008; Richter and Hargreaves 2013). This type of signaling redundancy may account for the fact that exercise can still increase skeletal muscle glucose uptake when that same muscle is insulin resistant.

A previous study demonstrated that in vitro knockdown (>95%) of PKC‐*λ* in myotubes was associated with enhanced insulin sensitivity and insulin‐stimulated glucose uptake (Stretton et al. 2010). In our current experiments, contrary to our hypothesis, we observed similar improvements in contraction‐mediated glucose uptake. Collectively these findings suggest that loss of aPKC activity results in general improvements in glucose uptake. M*λ*KO mice, however, did not demonstrate augmented glucose uptake compared to WT controls during treadmill running (Sajan et al. 2010). Differences in experimental methods, including exercise type (treadmill vs. contraction), experimental model (in vivo vs. in situ/in vitro) and studied muscle (vastus lateralis vs. TA and soleus), possibly underlie these observations.

In summary, contraction‐stimulated glucose uptake is not decreased with disruption of aPKCs. In contrast, loss of aPKC activity augmented contraction‐induced muscle glucose uptake, which is associated with increased resistance to muscle fatigue. Although we cannot rule out compensation mechanisms during loss of aPKC activity, our data and a previous study (Sajan et al. 2010) suggest aPKCs are not necessary for glucose uptake with exercise and contraction. Contractile activity stimulates multiple intracellular signaling mechanisms in skeletal muscle, and it is likely that there is redundant signaling for the regulation of glucose uptake.

## Conflict of Interest

None declared.
